# Medical students’ perceptions of an emergency medicine clerkship: an analysis of self-assessment surveys

**DOI:** 10.1186/1865-1380-5-25

**Published:** 2012-05-31

**Authors:** Jennifer L Avegno, Heather Murphy-Lavoie, Daryl P Lofaso, Lisa Moreno-Walton

**Affiliations:** 1 Section of Emergency Medicine, Louisiana State University Health Sciences Center, New Orleans, USA; 2 The Learning Center, Louisiana State University Health Sciences Center, New Orleans, USA

## Abstract

****Background**:**

No studies have been performed that evaluate the perceptions of medical students completing an emergency medicine (EM) clerkship. Given the variability of exposure to EM in medical schools nationwide, assessment of the student rotation may inform the structure and content of new and existing clerkships, particularly in relation to student’s acquisition of the core competencies.

****Objectives**:**

To investigate whether undergraduate medical students rotating through an EM clerkship improved their understanding and abilities in core content areas and common procedural skills; to evaluate whether improvement was affected by rotation length.

****Methods**:**

All students participating in an EM clerkship over a 12-month period were asked to complete an anonymous voluntary pre- and post-rotation survey. Confidence with patient assessment, diagnosis, and management plans; trauma and medical resuscitations; formal and informal presentations; basic procedure skills and understanding of the modern practice of EM were self assessed using a Likert scale. Group mean scores on each question on the pre- and post-clerkship surveys were calculated and compared. The mean scores on each survey item, both pre- and post-clerkship, were compared between 2- and 4-week clerkship rotation groups.

****Results**:**

Two hundred thirty-nine students participated in the rotation during the 12 months of the study. One hundred sixty-one (161), or 67.4%, completed the pre-rotation survey, and 96 (40.2%) completed the post-rotation survey. Overall, students showed significant mean gains in confidence with initial patient assessment, diagnosis, and management plans (*p* < 0.01, 0.02, <0.01) and with basic procedure skills (*p* < 0.01 for all). Students completing a 2-week rotation did not differ significantly from f4week rotators in confidence levels, except in the area of formal presentation skills (*p* = 0.01), where the 4-week students demonstrated a statistically significant advantage. The 2-week clerkship participants were significantly less confident in all procedures except EKG interpretation, splinting, and venipuncture (*p* = 0.28, 0.22, 0.05). Regardless of rotation length, students generally felt they had sufficient exposure to patients and opportunities for hands-on learning and practice, and overwhelmingly would recommend the EM clerkship to a fellow student, regardless of their chosen specialty.

****Conclusions**:**

Medical students show significant gains in confidence with acute care knowledge, disease management, and procedure skills after completion of an EM clerkship. Although a 4-week clerkship may be preferable to expose students to the widest variety of patients and procedures, all students can benefit and improve in core competencies after an EM undergraduate experience.

## **Background**

Medical student experience in emergency and acute care is considered to be a critical part of undergraduate medical education [1,2]. While the number of mandatory Emergency Medicine (EM) clerkships doubled between 1992 and 2005, fewer than one-half of US allopathic medical schools offer a dedicated EM rotation in their curriculum [3]. EM rotations contribute to student education by teaching basic and advanced resuscitation; approach to the undifferentiated patient; simple, common procedural skills, and increased understanding of EM as a specialty. However, EM clerkships vary in rotation length, total hours worked, shift length, and didactic exposure. Furthermore, little has been described in the literature about medical students’ experience during their EM rotations [4].

EM students have been surveyed about their learning preferences and perceived characteristics of good teachers [5]; however, no data exist on students’ own assessment of what they gain in clinical knowledge and procedural skills during an EM clerkship. All students, even those not applying for EM residency, should improve their abilities in the recognition and management of the undifferentiated patient, acute resuscitation, and basic procedural skills after successful completion of a junior- or senior-level EM rotation. While textbook knowledge can be easily evaluated using objective tests, many management and procedural skills are less conducive to objective assessment. Comparing students’ own perceptions of their abilities before and after an EM rotation may provide important data on skills actually gained during the experience and fulfillment of the clerkship objectives.

It has been suggested previously that standardizing the EM rotation may provide students with a consistent experience and improve performance [6,7]. At present, however, students are exposed to a wide variety of EM clerkship lengths and styles. The medical school curriculum is full of many different specialties all competing for dedicated time. While a senior 4-week rotation in EM may be ideal [8], students might also gain significant experience and exposure in core EM objectives during a shorter time period. Comparing the experience gained in a traditional 4-week clerkship with that in a rotation of shorter length may assist those departments wishing to implement or revise an EM curriculum and inform their efforts in requesting institutional approval and support.

This study investigated whether medical students completing an EM rotation improved their understanding and abilities in basic EM core content areas and common procedural skills, as evidenced by self-assessment reports. In addition, we compared the experiences of students rotating through a 2-week EM clerkship to those completing a 4-week clerkship to determine whether the length of rotation influences assessment of skills and knowledge gained during the experience.

## **Methods**

### **Study design**

This was a prospective observational study that was reviewed by our Institutional Review Board and met criteria for exempt status.

### **Study setting and population**

University Hospital in New Orleans is an academic, urban teaching hospital with an annual census of greater than 60,000 patient visits. The Emergency Department (ED) is staffed with board-certified EM physicians and is the primary training site for a PGY1-4 EM residency program with 52 residents. Medical students, EM residents, and interns from a variety of specialties staff the ED under the supervision of academic EM faculty. The ED serves as the sole location for two EM clerkships from nearby Louisiana State University (LSU) and Tulane University medical schools, as well as visiting students from other nationally and internationally accredited institutions. Both local schools consider University Hospital a primary training hospital for students and residents. Tulane students must take a mandatory 2-week EM rotation in their third or fourth year of medical school, whereas LSU and visiting students are offered only a 4-week elective in their fourth year.

All students worked approximately 36 h per week in 12-h shifts, equally distributed between day and overnight shifts The 2-week rotation offers two Fast Track (FT) and four Main Emergency Room (MER) shifts, while the 4-week rotation offers four FT shifts and eight MER shifts. All students attended 2 to 3 hours of didactic lectures and labs per week. Regardless of rotation length, students had the same patient care duties and expectations in the ED. All course requirements were identical with one exception: students rotating on the 4-week course were required to give a mandatory formal case presentation to EM clerkship directors and fellow students during their block, whereas 2-week rotators had no such requirement.

### **Study protocol**

Between July 2008 and June 2009, medical students enrolled in the EM clerkship were contacted prior to the start of their rotation and invited to participate in an anonymous online pre-rotation survey on their prior experience and present confidence with patient assessment, diagnosis, and management plans; trauma and medical resuscitations; formal and informal presentations; and specific procedure skills (listed in Table 1). These measures were crafted to reflect several of the Liaison Committee for Medical Education (LCME) core competencies, namely: patient care, medical knowledge, interpersonal skills, and systems-based practice [2]. Students were also queried about their interest in EM as a specialty.

**Table 1:** Comparison of self-assessed skills and abilities before and after an EM rotation for all participating students

	**Pre-test mean score**	**Post-test mean score**	*P*-value (*t*-test)*
Conduct an initial assessment of an acutely ill patient	3.60	4.19	<0.01
Identify a “sick” vs. “non-sick” patient	3.97	4.31	<0.01
Formulate a differential diagnosis	4.05	4.25	0.02
Develop an appropriate management plan	3.40	3.95	<0.01
Explain to others the core elements and principles of the modern practice of EM	2.68	3.83	<0.01
Present patient cases in a formal setting	3.42	4.12	<0.01
Present patient cases in an informal setting	3.94	4.42	<0.01
Interest in EM as a specialty†	2.59	2.59	.99
**Procedures:**			
Wound closure and repair	2.99	4.12	<0.01
Gynecologic exams	3.45	4.42	<0.01
Foley catheter insertion	3.22	3.93	<0.01
Incision & drainage	2.90	3.85	<0.01
EKG interpretation	2.65	3.76	<0.01
Basic life support	2.62	4.06	<0.01
Major medical resuscitation	1.77	3.16	<0.01
Major trauma resuscitation	1.65	3.52	<0.01
Splinting/fracture care	2.07	2.70	<0.01
IV access/venipuncture	2.44	2.65	<0.01
NG tube placement	2.07	3.09	<0.01

*P-values calculated using two-tailed *t*-test for samples of equal variance.

^ Scored using a Likert scale ranging from 1 (“not confident at all”) to 5 (“great confidence”).

†Scored using a Likert scale from 1 (“no interest at all”) to 5 (“great deal of interest”).

At the end of each rotation, both 2- and 4-week students were invited to take an anonymous online post-rotation questionnaire to assess their current level of confidence on the same measures. In both surveys, Likert scales were used ranging from 1 (“not confident at all”) to 5 (“great confidence”) to score questions about students’ level of confidence with the skills and procedures under study. Post-rotation questions on students’ perception of the sufficiency of clinical content (patient exposure, hands-on learning, ability to demonstrate knowledge) and usefulness of the rotation for students were scored 1 (strongly disagree) to 5 (“strongly agree”). Survey participation was voluntary. Surveys included no subject identifiers and did not count in any way towards students’ grades.

### **Data analysis**

Chi-square analysis was used to compute differences between proportions of 2-week vs. 4-week student responders. Mean responses for both pre- and post-test survey questions were calculated with 95% confidence intervals. Student’s *t*-test was used to calculate the difference between group mean pre- and post-rotation skill and procedure confidence levels. To assess whether length of rotation results in significantly different confidence in skills and abilities, the difference between 2- and 4-week student group means on the post-test was also calculated using Student’s *t*-test. A *p*-value of less than 0.05 was considered significant.

## **Results**

Two hundred thirty-nine students completed an EM rotation between July 2008 and June 2009. Of these, 110 (46%) participated in the 2-week course, and 129 (54%) took part in the 4-week rotation. Response rates were 67.4% for the pre-test and 40.2% for the post-test. Study participation rates were consistent between groups of students (see Table 2).

**Table 2:** Survey participation rates by type of rotation

	**Totalparticipating in rotation**	Total surveyrespondents—pre-test (%)*	Total survey respondents—post-test (%)*
2-Week students	110	79 (72)	47 (43)
4-Week students	129	82 (64)	49 (38)
Totals	239	161 (67)	96 (40)

*Non-significant (p > 0.05) differences between proportions of survey respondents and total participants for both 2- and 4-week students by chi-square analysis.

Overall, students rated significantly greater confidence with assessment, diagnosis, and management of the acutely ill patient after completing an EM clerkship (Table 1). Post-rotation scores improved for both formal and informal presentation skills, as well as understanding of the modern practice of EM. Students reported significant gains in confidence with basic common procedural skills after their rotation, particularly in the areas of medical and trauma resuscitation. However, there was no significant increase in interest in EM as a specialty after completing a rotation.

There were no significant differences in mean confidence levels between the 2- and t4-week rotation groups for patient assessment, diagnosis, and management, or informal case presentation (Table 3). However, the 4-week students reported a significantly higher mean confidence level with formal case presentation, likely due to this requirement in the longer clerkship. Four-week rotators also reported higher confidence levels with most of the procedures surveyed. Only EKG interpretation, splinting/fracture care, and venipuncture—all very common student procedures in our ED—showed no significant differences in confidence level between 2- and 4-week rotators.

**Table 3:** Comparison of self-assessed skills and abilities after an EM rotation by length of clerkship

	**Two-week post-test mean score**	**Four-week post-test mean score**	*P*-value(*t*-test)*
Conduct an initial assessment of an acutely ill patient	4.17	4.21	0.79
Identify a “sick” vs. “non-sick” patient	4.30	4.32	0.88
Formulate a differential diagnosis	4.23	4.26	0.83
Develop an appropriate management plan	3.91	3.98	0.57
Explain to others the core elements and principles of the modern practice of EM	3.74	3.91	0.33
Present patient cases in a formal setting	3.89	4.33	0.01
Present patient cases in an informal setting	4.34	4.50	0.25
**Procedures:**			
Wound closure and repair	3.64	4.21	0.00
Gynecologic exams	3.53	4.17	0.00
Foley catheter insertion	3.28	4.23	0.00
Incision & drainage	3.77	4.35	0.00
EKG interpretation	3.06	3.25	0.28
Basic life support	3.28	3.75	0.03
Major medical resuscitation	2.48	2.92	0.03
Major trauma resuscitation	2.40	2.90	0.02
Splinting/fracture care	2.96	3.23	0.22
IV access/venipuncture	2.87	3.27	0.05
NG tube placement	2.51	3.13	0.01

*P-values calculated using two-tailed *t*-test for samples of equal variance.

^ Scored using a Likert scale ranging from 1 (not confident at all) to 5 (great confidence).

Mean scores of satisfaction with patient care variety and opportunities for skill development and practice were consistent for both the 2- and the 4-week rotations (Table 4). Students completing the longer rotation were significantly more likely to agree that the variety of patient presentations (mean Likert scores of 4.58 vs. 4.24 for the 2-week group) and opportunities for hands-on learning (mean scores of 4.49 vs. 4.02) were sufficient. However, both groups showed similar levels of agreement with the adequacy of available opportunities to demonstrate what they had learned (mean scores of 4.04 vs. 4.23, *p* = 0.34). Overall, respondents agreed that both students interested in EM as a career and those choosing other specialties should enroll in the EM clerkship (94.6% vs. 88.3%, respectively).

**Table 4:** Student assessment of experience by rotation length

	**Two-week post-test mean score**	**Four-week post-test mean score**	*P*-value (*t*-test)*
I was involved in a sufficient variety of patient presentations and conditions to enhance my learning	4.24	4.58	0.02
I had sufficient opportunities for hands-on learning and practice	4.02	4.49	0.01
I had sufficient opportunities to demonstrate my abilities and what I learned	4.04	4.23	0.34
I had sufficient experiences in the clerkship to learn about the practice of Emergency Medicine	4.04	4.40	0.02

*P-values calculated using two-tailed *t*-test for samples of equal variance.

^ Scored using a Likert scale ranging from 1 (strongly disagree) to 5 (strongly agree).

## **Discussion**

Standardizing and optimizing the student clinical experience in EM has long been a goal of educators. It is generally accepted that an EM rotation can provide exposure to clinical experiences rarely found elsewhere in undergraduate medical education: acute undifferentiated patient care, resuscitation and procedural skills, health care system management, and unique topics such as pre-hospital care and disaster training [3,8-11]. Indeed, LCME guidelines state that undergraduate educational opportunities “must be available in multidisciplinary content areas, such as Emergency Medicine” [2]. Researchers have suggested that EM clerkship curricula and core objectives can be readily adapted to reflect the LCME core competencies [12], yet data are lacking on the effect such a curriculum has on student experience.

Our study assessed students’ self-reported abilities in several of the core competencies: patient care (determining sick vs. non-sick patients; initial patient assessment; procedure skills); medical knowledge (developing differential diagnoses and management plans); interpersonal skills (formal and informal presentations); and systems-based practice (understanding and being able to explain the practice of EM). Regardless of rotation length, students reported high levels of perceived skills and knowledge after finishing the EM clerkship. Overall, mean confidence levels improved significantly on all measures, indicating that not only can core competencies be integrated into an EM clerkship, but students are likely to increase their understanding of and confidence with these critical concepts during an EM rotation.

Other self-assessment EM studies have used focus groups or self-recorded patient encounter and procedure logs to describe students’ experiences [5-7]. A study of preclinical students on an EM observership found that early exposure to EM was perceived as a worthwhile experience and encouraged participants to pursue other EM rotations in the clinical years [13]. Comparison of an EM with an Internal Medicine (IM) clerkship revealed that EM students were more likely to be involved in the initial assessment, diagnosis, management, and procedural intervention of their patients than were their IM counterparts [14]. Data on self-assessment of students’ procedural skills over the third year of medical school found that although students reported increased self-confidence in basic procedures over a 1-year timeframe, many had never participated in critical tasks such as venipuncture, cardioversion, or CPR [15]. Our results indicate that students gain significant confidence in both knowledge-based and manual skills during an EM clerkship, and are exposed to a wide variety of patients and tasks. This would seem to be far more desirable than the often erratic opportunities for acquisition of acute and emergent care competencies in the clinical years on other rotations.

Our study further demonstrated that although students in a traditional 4-week senior EM clerkship had generally higher mean post-rotation procedural scores, clinical and diagnostic abilities were equally high with 2-week rotators. Both groups of students reported strong levels of confidence with acute care assessments, management, diagnosis, and understanding of EM as a specialty. Nationally, medical school curricula vary considerably, and time allotted to EM may be limited based on competing demands of specialties that have been traditionally regarded as more essential to medical education. Previous research and American College of Emergency Physicians (ACEP) guidelines recommend integration of EM education throughout all 4 years of medical school with increasing length of exposure as students advance [8,10]. Our results indicate that even a shorter rotation provides rotators with enhancement of their clinical and procedural abilities, though perhaps 2 weeks is not sufficient time to learn and improve a great variety of basic procedural skills. Ideally, a 2-week rotation could initiate a medical student into acute care and the practice of EM, and may be followed by a longer clerkship to deepen core knowledge and build further confidence.

Interestingly, we found that exposure to an EM rotation does not increase interest in the specialty. This is consistent with previous data from medical schools with third-year EM clerkships [16] and may be due to the fact that many students have decided their specialty of choice prior to their third (and certainly fourth) year of study. Although there is a growing need for an increased number of EM physicians and one of the objectives of an EM clerkship should be to introduce all students to our specialty, every student can gain from experience in the ED. For students choosing other careers, this may be their last opportunity to care for patients outside their chosen specialties, and it should educate them on the role of EM and how their field interacts with the ED [17]. Our data indicate strong student support for participating in an EM clerkship, regardless of the chosen specialty. This support may be useful for clerkship directors and academic faculty when advocating for a new or expanded EM rotation at their institution, as medical school curriculum committees and administrators are often quite receptive to feedback from students about their educational goals and experiences [8].

### **Limitations**

Several features of this study may limit the generalizability of the results. First, response rates were relatively low and varied between pre- and post-rotation surveys. Since the surveys were both fully voluntary, it may be that busy or uninterested students did not take the time to respond, particularly after the clerkship was complete. However, given that an entire class of medical students at Tulane and the majority of senior LSU students participated in an EM rotation, we feel that the students are of sufficient variety to provide relevant data. We did not extend the survey to other specialties’ clerkships, which may also limit results; however, a recent study found that student clerkship self-assessment results varied widely and were caused by different factors depending on the specialty [18].

Because there were students from two different institutions, it may be that certain features of their home schools—such as previous exposure to EM or presence of a strong EM department or residency program—influenced their attitudes towards the specialty and thus introduced unmeasured variability into the results. Similarly, selection bias may confound our findings: since the 2-week rotation was a mandatory one and the 4-week clerkship was elective, students choosing the course may have been more likely to report a positive experience or improvement in skills. A previous study has suggested that elective students have made a conscious decision to study EM, whereas those on mandatory rotations may have varying levels of interest in the field, but both can gain from the clerkship experience [19]. Regardless of mandatory or elective status, over 85% of our respondents agreed that EM and non-EM bound students should enroll in the clerkship, which supports the conclusion that all students can benefit from the experience.

## **Conclusions**

Based on self-assessment reports, students show significant gains in confidence with acute care knowledge, disease management, and procedure skills after completion of an EM clerkship. Although 4 weeks may be preferable for exposing students to the widest variety of patients and procedures, all students can benefit and progress in the core competencies. Students agree that the EM rotation experience is beneficial for themselves and for their colleagues, regardless of future career choice. Further research should focus on linking self-assessed abilities to directly observed abilities, and in refining a standard set of core objectives and procedure skills for application in any EM rotation setting.

### *Open Access*

This article is distributed under the terms of the Creative Commons Attribution License, which permits any use, distribution, and reproduction in any medium, provided the original author(s) and source are credited.
